# Early Intensified Rehabilitation Training with Hyperbaric Oxygen Therapy Improves Functional Disorders and Prognosis of Patients with Traumatic Brain Injury

**DOI:** 10.1089/wound.2018.0876

**Published:** 2021-09-21

**Authors:** Yin Lu, Xianshan Zhou, Jincheng Cheng, Qing Ma

**Affiliations:** ^1^ College of Biology and Environmental Engineering, Zhejiang Shuren University, Hangzhou, China.; ^2^ Traumatic Rehabilitation Center of Hangzhou Sanatorium, Hangzhou, China.; ^3^ Departments of Neurology and Surgery, Bengbu, China.

**Keywords:** traumatic brain injury, hyperbaric oxygen therapy, comprehensive rehabilitation, function assessment, multicenter research

## Abstract

**Objective:** Traumatic brain injury (TBI) is a global public health problem. Hyperbaric oxygen (HBO) therapy may be beneficial for TBI because it improves cerebral blood flow into tissues exhibiting low blood flow. This was done to observe the clinical therapeutic effect of different intensities of rehabilitation training and HBO therapy in early stages of TBI.

**Approach:** In this multicenter, randomized, stratified case-controlled prospective clinical trial, we selected 158 patients with moderate-severe TBI and assigned them into (1) a control group receiving routine once-daily (1/d) rehabilitation training without HBO, (2) study group A receiving routine 1/d rehabilitation training with HBO, (3) study group B receiving twice-daily (2/d) intensified rehabilitation training with HBO, and (4) study group C receiving 2/d intensified rehabilitation training without HBO, all for 3 months. The cognitive ability, activities of daily life (ADL), and movement ability were assessed before and after training with the Fugl-Meyer Assessment (FMA), Functional Independence Measure (FIM), Modified Barthel Index (MBI), and Mini-Mental State Examination (MMSE).

**Results:** FIM, FMA, MBI, and MMSE scores were improved significantly after 1-, 2-, and 3-month rehabilitation training in all TBI patients (*p* < 0.01), and this improvement was especially remarkable in patients who received 2/d intensified rehabilitation training with HBO (*p* < 0.01).

**Innovation:** With extensive and intensive research on TBI rehabilitation, it was proved that TBI rehabilitation intervention should be initiated as early as possible.

**Conclusion:** Early intensified rehabilitation training in combination with HBO is more beneficial to the recovery of cognitive, ADL, and movement abilities of TBI patients.

**Figure:**
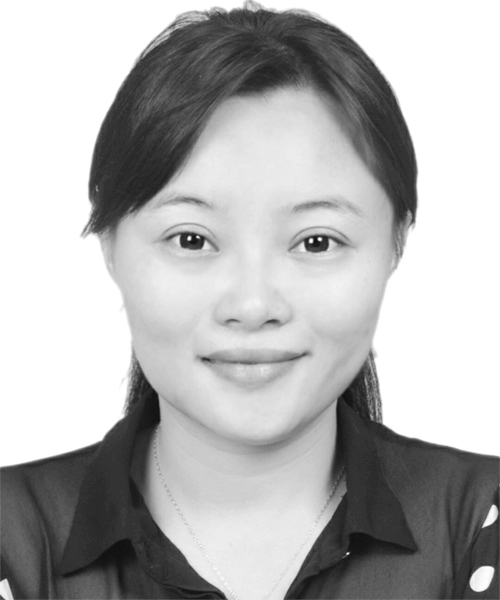
Yin Lu, PhD

## Introduction

Traumatic brain injury (TBI) refers to blunt, penetrating, or acceleration/deceleration force-derived craniocerebral injury, which causes symptoms such as decline in level of awareness or consciousness, memory loss or forgetfulness, other neurological or neuropsychological abnormalities, and even death.^1^ It is a global public health problem associated with high socioeconomic costs and substantial loss of healthy life-years due to ill health, disability, and/or early death.^2^ Cognitive and motor dysfunctions are the main sequelae in many patients with severe TBI and seriously affect the quality of life of these patients.^3^ In recent years, more attention has been paid to physiological, psychological, and cognitive rehabilitation of patients with moderate-severe TBI.^4,5^ With extensive and intensive research on TBI rehabilitation, it is generally accepted that TBI rehabilitation intervention should be initiated as early as possible. Rehabilitation initiated within 1 month after TBI is called early rehabilitation.^6^ Experimental studies have shown that initiation of rehabilitation intervention in the acute stage could improve the functional disturbances of rats with TBI.^7,8^ Clinical studies have also demonstrated that early rehabilitation is especially beneficial to functional improvement of patients with moderate-severe TBI, shortens the length of hospital stay, and reduces hospital expenses.^9,10^ On the contrary, delayed rehabilitation will unfavorably affect future functional recovery. Early rehabilitation has gradually achieved consensus in the circle of modern rehabilitation medicine. The intensity of rehabilitation training is an important factor influencing the therapeutic outcome. Previous studies have shown that intensified rehabilitation training can help TBI patients recover their functions sooner, improve their Glasgow Outcome Score and Functional Independence Measure (FIM) scores, and shorten the length of hospital stay markedly.^11–13^

Hyperbaric oxygen (HBO) therapy is intermittent inhalation of 100% oxygen at greater than normal atmospheric pressure and may be beneficial for TBI because it improves cerebral blood flow into tissues exhibiting low blood flow.^14^ Hyperoxia improves cerebral metabolic consumption of oxygen, and also leads to the improvement of oxygenation and reduction in cerebral edema and intracranial pressure.^15,16^ Usually, HBO therapy lasts for 6 months as one course and is generally followed up for 3–5 years. Clinically, HBO therapy can effectively improve the histological structure and brain function (significantly increasing regional cerebral blood flow) in varying degrees throughout the whole treatment period of TBI (it will be 6 months in general according to American college of surgeons trauma quality improvement program) and promote regeneration and remodeling of neurons (activating of redundant neurons and leading to significant neurological improvement).^17–19^

However, most of these studies on HBO therapy of TBI patients were limited to the chronic stages (at least 3 years postinjury with persistent neurological and brain deficits), and few studies have focused on the impact of HBO in the early stage of TBI. It is therefore unclear whether early HBO in combination with rehabilitation training could favorably affect functional recovery of TBI patients.

To observe the effect of different intensities of rehabilitation training in combination with HBO on early TBI, we conducted a multicenter randomized controlled prospective study in TBI patients in an attempt to explore the therapeutic efficacy of different-intensified rehabilitation training in combination with HBO on early TBI. Our hypothesis was that twice-daily (2/d) intensified rehabilitation training would be more beneficial for improving post-TBI dysfunctions when compared with the conventional once-daily (1/d) one. In addition, HBO may also promote functional recovery of TBI patients in early rehabilitation therapy. And the safety of 2/d intensified rehabilitation training was also explored and discussed.

## Clinical Problem Addressed

Knowledge of the effect of different intensities of rehabilitation training in combination with HBO on early TBI will be an important factor influencing the therapeutic outcome.

## Materials and Methods

### Definitions and classifications

TBI is defined as damage to the brain resulting from external mechanical force, such as rapid acceleration or deceleration, impact, blast waves, or penetration by a projectile. As a consequence of the injury, brain function is temporarily or permanently impaired and structural damage may or may not be detectable with current imaging technology. TBI is usually classified based on severity, anatomical features of the injury, and the cause of the injury. The severity is assessed according to the loss of consciousness duration, post-traumatic amnesia, and Glasgow coma scale (GCS) grading of the level of consciousness.^20^

### Samples

Included in this study were TBI patients who were admitted for treatment in No. 128 Hospital, No. 117 Hospital, No. 123 Hospital, and Hangzhou Hospital between January 2013 and December 2017. These hospitals are public hospitals with special trauma rehabilitation centers. Inclusion criteria were as follows: (1) TBI was confirmed by computed tomography (CT) or magnetic resonance imaging brain imaging, and the duration of TBI was less than 15 days, (2) GCS score ≤12 (severe form ≤8, moderate form 9–12), (3) age 18–70 years, and (4) no regular rehabilitation therapy before admission. Exclusion criteria were as follows: (1) history of motor and cognitive dysfunctions (elicited from patient and/or family), (2) presence of severe major organ deterioration or failure, (3) being able to take care of daily life with a Modified Barthel Index (MBI) >70, (4) persistent coma >15 days, and (5) inability to obtain consent from patient or/and family. A total of 179 TBI patients met these inclusion and exclusion criteria, and 21 were lost to follow-up. Finally, 158 TBI patients were enrolled in this study. According to the frequency and intensity of rehabilitation training with or without HBO therapy, patients were randomized to four groups: (1) the control group, in which patients received routine 1/d rehabilitation training without HBO; (2) study group A, in which the patients received routine 1/d rehabilitation training with HBO; (3) study group B, in which the patients received intensified 2/d rehabilitation training with HBO; and (4) study group C, in which the patients received intensified 2/d rehabilitation training without HBO. There was no significant difference in general clinical data (e.g., number of case and sex, age, and GCS score) among the four groups (*p* > 0.05), and therefore they were comparable, which facilitated the objective evaluation of the later experiment (Table 1). The research protocol was approved by the ethics committees of the related hospitals, and informed consent was obtained from all the participating patients' families before initiation of the study.

**Table 1.** General clinical data of the traumatic brain injury patients in four groups

Group	Case	Sex		Age (year)	GCS Score		Cause of Injury			
		M	F		≤8	9–12	Traffic Accidents	Falls from Height	Blunt Injury	Falls
Control	42	35	7	46.21 ± 10.45	26	16	25	14	0	3
Study group A	39	35	4	45.78 ± 11.24	27	12	23	11	1	4
Study group B	39	36	3	45.27 ± 13.72	26	13	24	9	2	4
Study group C	38	34	4	44.81 ± 12.68	25	13	26	7	3	2

GCS, Glasgow coma scale.

### Treatment methods

There was no significant discrepancy in emergency treatment measures during the acute stage among the four groups of patients. In addition to routine surgical and medical interventions and nursing care, patients in the control group received systematic and standardized functional training, including placement of the unaffected limb, functional electric stimulation (20 min at a time daily), acupuncture treatment (30 min at a time daily), exercise therapy (45 min at a time daily), occupational therapy (30 min at a time daily), speech therapy (30 min at a time daily), and cognitive behavior therapy (30 min at a time daily), using 1 month as a course of treatment for three courses. The training was conducted twice a day for patients in study group B and C. Patients in study group A and B also received HBO at 2.0 atmospheric pressure absolute (ATA) for 1 h daily for 20 days as a course of treatment for three courses, with a 10-day interval between every two courses.

### Evaluation methods

Each center appointed designated professionals (*n* = 3) to perform evaluation in a blind manner before initiation of the rehabilitation training after admission (T0), and at 1 (T1), 2 (T2), and 3 (T3) months after rehabilitation training, with Mini-Mental State Examination (MMSE), MBI, FIM, and Fugl-Meyer Assessment (FMA).

### Statistical analysis

Data were treated with EXCEL 2003 were analyzed statistically with SAS8.1 and are expressed as mean ± SD ($\bar{x}$ ± S). Chi-square test was used for counting data, and two-factor repeated measurement analysis of variance was used for measuring data. *p-*Values <0.05 were considered statistically significant.

## Results

### Impact of rehabilitation therapy on MBI of TBI patients

**Figure 1.**
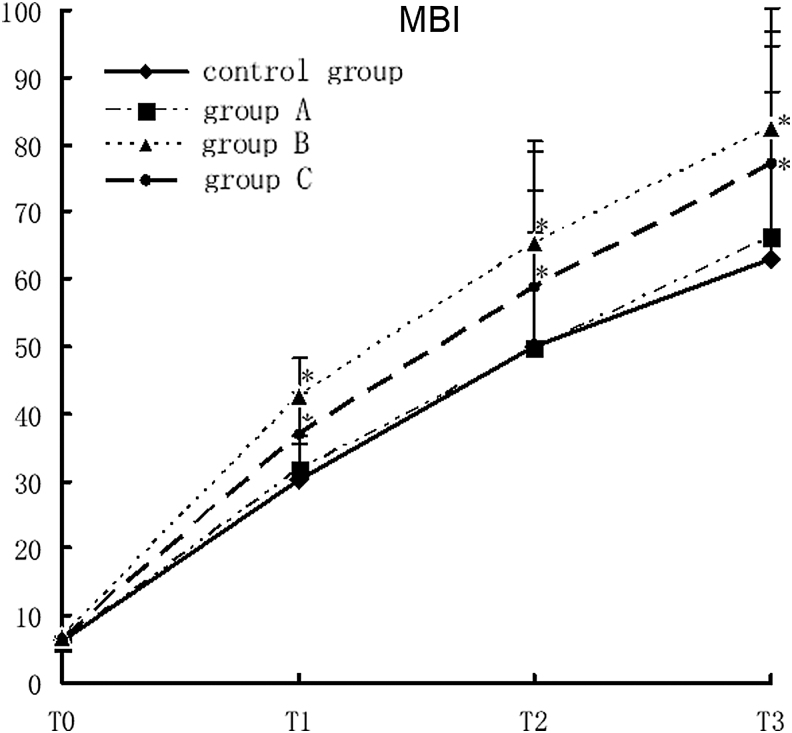
MBI scores of different groups with different rehabilitation interventions, compared with the control group, *p < 0.05. MBI, Modified Barthel Index.

The MBI scores in study group B and C were significantly higher than those in the control group at T1, T2, and T3 (*F* = 5.08, *p* < 0.0001). There was no significant difference in MBI between study group A and the control group at all designated time points. In addition, there was a cross-effect among different groups and time points (*F* = 5.02, *p* < 0.0001), suggesting that the MBI scores were not all the same in different groups and at different time points, nor were the rates of variance. Comparison of the MBI scores at different time points showed that there were statistically significant differences in MBI among different time points. With time prolonged, the MBI score in each group increased gradually. Comparison of the four time points showed T3 >T2 >T1 >T0 (*F* = 1309.71, *p* < 0.0001; Fig. 1).

### Impact of rehabilitation therapy on MMSE of TBI patients

**Figure 2.**
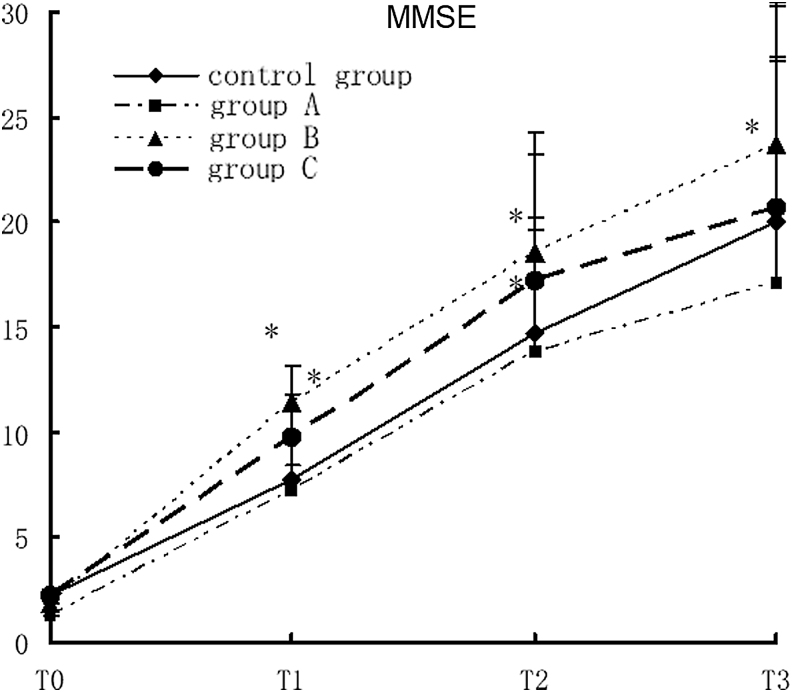
MMSE scores in different groups with different rehabilitation interventions, compared with the control group, *p < 0.05. MMSE, Mini-Mental State Examination.

The MMSE score in study group B was significantly higher than that in the control group at T1, T2, and T3. The MMSE score in study group 3 significantly increased at T3 (*F* = 3.99, *p* < 0.0003). The MMSE was relatively lower in study group A and the control group at all points. There was no significant difference in MMSE between the two groups at all points except T3. There was a cross-effect between different groups and different time points, suggesting that the MMSE scores were not all the same in different groups and at different time points, nor were the rates of variance (*F* = 5, *p* < 0.0001). Comparison of the MMSE scores at different time points showed that there were statistically significant differences in MMSE among different time points. With time prolonged, the MMSE score in each group increased gradually. Comparison of the four time points showed T3 >T2 >T1 >T0 (*F* = 908.86, *p* < 0.0001; Fig. 2).

### Impact of rehabilitation therapy on FIM of TBI patients

**Figure 3.**
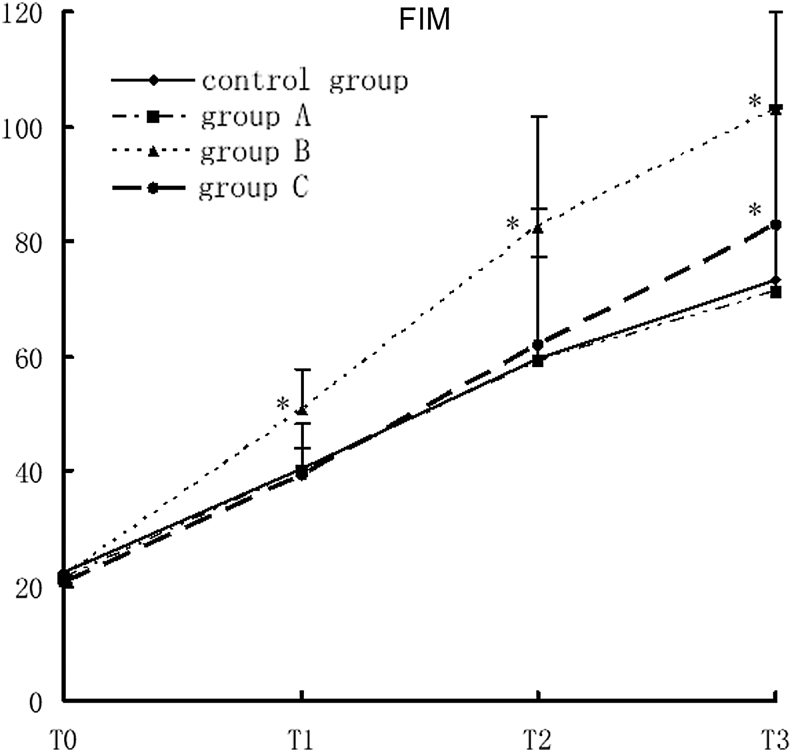
FIM scores in different groups with different rehabilitation interventions, compared with the control group, *p < 0.05. FIM, Functional Independence Measure.

The FIM score in study group B was significantly higher than that in the control group at T1, T2, and T3. The FIM score in study group 3 significantly increased at T3 (*F* = 5.91, *p* < 0.0001). There was no significant difference in FIM between the two groups at all points. There was a cross-effect between different groups and different time points, suggesting that the FIM scores were not all the same in different groups and at different time points, nor were the rates of variance (*F* = 6.53, *p* < 0.0001). Comparison of the FIM scores at different time points showed that there were statistically significant differences in FIM among different time points. With time prolonged, the FIM score increased gradually in all groups. Comparison of the four time points showed T3 >T2 >T1 >T0 (*F* = 874.68, *p* < 0.0001; Fig. 3).

### The impact of rehabilitation therapy on FMA of TBI patients

**Figure 4.**
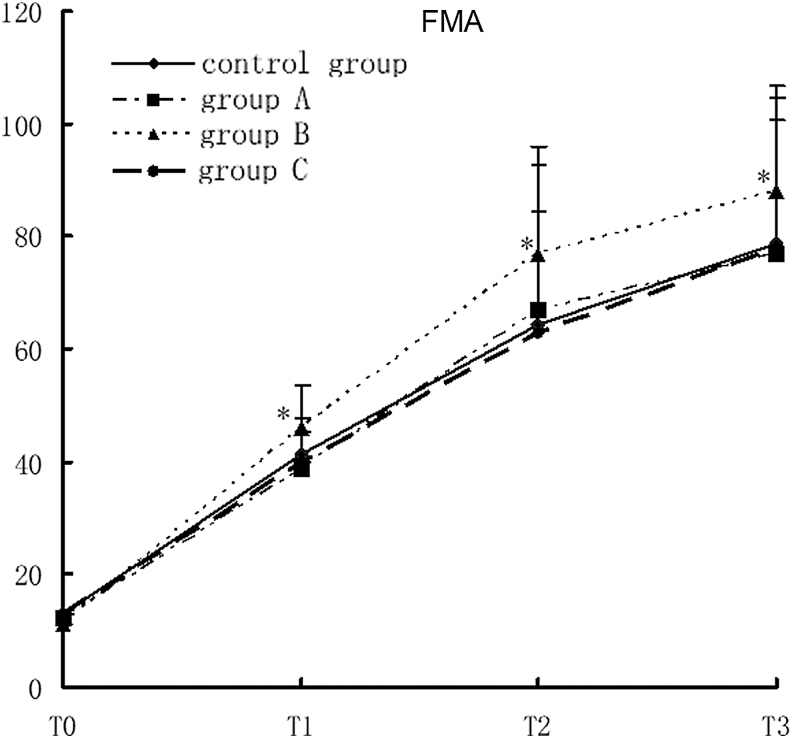
FMA scores in different groups with different rehabilitation interventions, compared with the control group, *p < 0.05. FMA, Fugl-Meyer Assessment.

The FMA score in study group B was significantly higher than that in the control group at T1, T2, and T3 (*F* = 5.69, *p* < 0.0001). There was no significant difference in FMA among study group A and C and the control group at all designated time points. There was a cross-effect between different groups and different time points (*F* = 5.56, *p* < 0.0001), suggesting that the FMA scores were not all the same in different groups and at different time points, nor were the rates of variance. Comparison of the FMA scores at different time points showed that there were statistically significant differences in FMA among different time points. With time prolonged, the FMA score increased gradually in all groups. Comparison of the four time points showed T3 >T2 >T1 >T0 (*F* = 1390.93, *p* < 0.0001; Fig. 4).

## Discussion

The results of this study are consistent with our initial expectation. Compared with the control group, intensified rehabilitation training was able to improve post-TBI functional disorders. Intensified rehabilitation training in combination with HBO therapy could further improve post-TBI functional disorders. However, 1/d rehabilitation training in combination with HBO did not significantly improve the cognitive ability, movement ability, or activities of daily life (ADL) of the TBI patients. The results of this study indicate that TBI patients were able to tolerate 2/d rehabilitation training without sustaining significant adverse effects and the rate of giving up and missing the training was relatively lower, suggesting that the 2/d intensified rehabilitation training is a safe and feasible regimen.

The present study also found that MBI, MMSE, FIM, and FMA scores of the TBI patients increased gradually with the lapse of time during the 3-month systematic, standardized, and comprehensive rehabilitation training in all groups, indicating that early, sustainable comprehensive rehabilitation training can help TBI patients improve their ADL and cognitive ability and promote the recovery of mobility of their extremities. These findings are consistent with the reports in the literature.^13,21,22^

Our study found that after 1 month of training, MBI and MMSE scores of the patients in the intensified training groups began improving significantly as compared with the control group, while there was no significant improvement in FIM or FMA. Compared with that of the control group, MBI, MMSE, FIM, and FMA in the intensified rehabilitation training + HBO group were significantly better and were not significantly different between the routine training + HBO and control groups, indicating that intensified training can improve ADL and cognitive ability, especially movement ability. These effects became more obvious after 2- and 3-month rehabilitation training, especially in the intensified rehabilitation training groups. There was no significant difference in FIM, MBI, or FMA between the routine training and control groups, indicating that the therapeutic outcome of HBO is closely correlated with the intensity of rehabilitation training. However, there are studies reporting that HBO did not help much in improving the movement and cognitive abilities of TBI patients.^23,24^ Unlike our study, these studies explored the effect of HBO simply from the aspect of HBO itself without taking rehabilitation training into account. Our opinion is that TBI rehabilitative intervention should be comprehensive by using multiple therapeutic methods. We found in our study that HBO therapy could partially improve post-TBI functional disorders, and this effect was closely correlated with the intensity of rehabilitation training. We maintain that HBO should be combined with rehabilitation training, and it can work only when the training reaches a certain degree of intensity. In addition, in a previous study, HBO was reported to have some adverse and toxic effects.^25^ Some patients experienced severe ear pain and received tympanostomy. Thereafter, the ear pain subsided, and the patients completed the full course of HBO therapy successfully. However, in our practice of using 2.0 ATA HBO × 60, we did not find significant adverse effects, indicating that HBO is relatively safe, which is consistent with some other previous studies.^1,25,26^ Although Lin *et al.* reported that using 2.0 ATA HBO for the treatment of subacute moderate/severe TBI induced occurrences of epilepsy in about 9% patients,^27^ we did not encounter such a case in our study.

We maintain that HBO should be combined with rehabilitation training, and it can work only when the training reaches a certain degree of intensity. The improved tissue oxygenation and cellular metabolism, antiapoptotic and anti-inflammatory effects may constitute the multiple and complementary mechanisms underlying HBO-induced neuroprotection.^28^ The brain receives 15% of the cardiac output, consumes 20% of the total body oxygen, and utilizes 25% of the total body glucose. Still, this energy supply is only sufficient to keep about 5–10% of the neurons active at any given time. Thus, at standard healthy condition, at any given time, the brain is utilizing almost all oxygen/energy delivered to it. Indeed, the intensified rehabilitation training process after brain injury requires much additional energy. This is where HBO treatment can help the increased oxygen level in the blood and body tissues during treatment can supply the energy needed for brain repair.^20^ Thus, HBO therapy may serve as a promising neuroprotective strategy that when combined with other therapeutic targets for TBI patients could improve long-term outcomes.

There are some limitations to this study. For instance, this study did not include patients with extremely severe TBI, and all the included patients were highly cooperative and had a strong willingness to participate in the training, which lead them to the completion of the whole training program and the achievement of ideal outcome. And, further research of a subanalysis could be made to try and find out whether HBO was necessary equally all along 1-, 2-, and 3 months.

We must admit that the results of HBO therapy in clinical TBI trials are currently controversial. There are four main reasons. First, the optimal time window for HBO administration is one of the crucial facts that determine its efficacy in TBI. The data of these preclinic and clinic studies indicated that HBO is beneficial when it is applied in the early stage.^29^ Application of HBO therapy within a therapeutic time window established in preclinical study is an important requirement for efficiency. Second, objective and precise assessment methods are another challenge in the evaluation of the efficacy of HBO therapy in TBI patients. Cognitive, emotional, behavioral, and physical impairments are common sequelae of TBI. Relying totally on the self-administration assessments is a weakness of these studies. In most clinical studies, more objective assessment methods, such as brain single photon emission CT imaging or electrophysiological measurement may be needed to provide authentic evidences for HBO therapy or control interventions and to allow a greater refinement of HBO therapy in TBI. Third, heterogeneity of the patients and HBO paradigms (pressure, frequency, and length of treatment course) partly affect or determine the outcome. There were variations in age of patients, and in severity and nature of the injury in the studies. There is significant preference that HBO showed more efficiency in younger age.^30^ Fourth, HBO therapy is not a completely benign process and there are concerns about its safety aspects. Possible complications during HBO therapy include barotraumatic lesions (middle ear, nasal sinuses, inner ear, lung, and teeth), oxygen toxicity (central nervous system and lung), confinement anxiety, and ocular effects (myopia and cataract growth).^31^ In conclusion, if safety guidelines are strictly followed, HBO therapy is a modality with acceptable rate of complications.

## Innovation

It was found in our study that intensified rehabilitation training could not only improve the ADL and cognitive ability of TBI patients but also improve their prognosis, because movement training can obviously promote the plasticity of the central nervous system's structure and function.^32–36^ We suppose that the role of intensified rehabilitation training in improving the functional disorders of TBI patients may be associated with its effect of promoting nerve plasticity. Early intensified rehabilitation training in combination with HBO is more beneficial to the recovery of cognitive, ADL, and movement abilities of TBI patients.

**Key Findings**

- Intensified rehabilitation training in combination with HBO therapy could further improve post-TBI functional disorders.
- The early, sustainable comprehensive rehabilitation training can help TBI patients improve their ADL and cognitive ability and promote the recovery of mobility of their extremities.
- HBO should be combined with rehabilitation training, and it can work safely only when the training reaches a certain degree of intensity.

## About the Authors

**Lu Yin, PhD**, is Professor of Medicine at Zhejiang Shuren University where she studies oxygen and hypoxia in clinical cases. **Zhou Xianshan, MD**, is head of a research group at Traumatic Rehabilitation Center of Hangzhou Sanatorium No. 128 Hospital of China with a focus on traumatic brain injury research. **Cheng Jincheng, MD**, is an orthopedic trauma surgeon at Departments of Neurology and Surgery of No.123 Hospital of China. **Ma Qing, PhD,** is a research fellow in Dr. Lu Yin's Laboratory at Zhejiang Shuren University.

## Abbreviations and Acronyms

1/d: once-daily
2/d: twice-daily
ADL: activities of daily life
ATA: atmospheric pressure absolute
CT: computed tomography
FIM: functional independence measure
FMA: fugl-meyer assessment
GCS: glasgow coma scale
HBO: hyperbaric oxygen
MBI: modified barthel index
MMSE: mini-mental state examination
TBI: traumatic brain injury

## References

[1] Baoqi D, Wenli C, Weichun H, Gang C. Rehabilitation treatment and progress of traumatic brain injury dysfunction. Neural Plast 2017;2017:1582182. 28491478 10.1155/2017/1582182 PMC5405588

[2] Andelic N. The epidemiology of traumatic brain injury. Lancet Neurol 2013;12:28–29. 23177533 10.1016/S1474-4422(12)70294-6

[3] Zhang GX, Zhang F, Zhang T, et al. Tetramethylpyrazine nitrone improves neurobehavioral functions and confers neuroprotection on rats with traumatic brain Injury. Neurochem Res 2016;41:2948–2957. 27452038 10.1007/s11064-016-2013-y

[4] Kramer ME, Suskauer SJ, Christensen JR, et al. Examining acute rehabilitation outcomes for children with total functional dependence after traumatic brain injury: a pilot study. J Head Trauma Rehabil 2013;28:361–370. 22613944 10.1097/HTR.0b013e31824da031 PMC3470756

[5] Mauritz W, Wilbacher I, Leitgeb J, et al. One-year outcome and course of recovery after severe traumatic brain injury. Eur J Trauma and Emerg Surg 2011;37:387–395. 26815275 10.1007/s00068-010-0053-6

[6] Novack TA, Bush BA, Meythaler JM, Canupp J. Outcome after traumatic brain injury: pathway analysis of contributions from premorbid, injury severity, and recovery variables. Arch Phys Med Rehabil 2001;82:300–305. 11245749 10.1053/apmr.2001.18222

[7] Lippert-Grüner M, Mägele M, Svestková O, et al. Rehabilitation intervention in animal model can improve neuromotor and cognitive functions after traumatic brain injury: pilot study. Physiol Res 2011;60:367–375. 21114367 10.33549/physiolres.931816

[8] Itoh T, Imano M, Nishida S, et al. Exercise inhibits neuronal apoptosis and improves cerebral function following rat traumatic brain injury. J Neural Transm 2011;118:1263–1272. 21442353 10.1007/s00702-011-0629-2

[9] Ovalie F, Xu L, Pearson WS, Spelke B, Sugerman DE. Outcomes of pediatric severe traumatic brain injury patients treated in adult trauma centers with and without added qualifications in pediatrics-United States, 2009. Inj Epidemiol 2014;1:15. 27747674 10.1186/2197-1714-1-15 PMC5005579

[10] Norup A, Egeland J, Løvstad M, et al. Education, training, and practice among nordic neuropsychologists. Results from a professional practices survey. Clin Neuropsychol 2017;31:1–22. 10.1080/13854046.2017.1291857 28361565

[11] Zhu XL, Poon WS, Chan CH, Chan SH. Does intensive rehabilitation improve the functional outcome of patients with traumatic brain injury? Interim result of a randomized controlled trial. Br J Neurosurg 2001;15:464–473. 11813997 10.1080/02688690120097688

[12] Stephanie H. Effectiveness of physiotherapy and occupational therapy after traumatic brain injury in the intensive care unit. Crit Care Res Pract 2012;2012:768456. 22550570 10.1155/2012/768456 PMC3328889

[13] Turner-Stokes L, Pick A, Nair A, Disler PB, Wade DT. Multi-disciplinary rehabilitation for acquired brain injury in adults of working age. Cochrane Database of Syst Rev 2016;33:434–436. 10.1002/14651858.CD004170.pub2 16034923

[14] Tolga C, Bital B, Kemal K, et al. A case of tension pneumothorax during hyperbaric oxygen therapy in an earthquake survivor with crush injury complicated by ARDS (adult respiratory distress syndrome). Undersea Hyperb Med 2015;42:9–13. 26094299

[15] Sahni T, Jain M, Prasad R, Sogani SK, Singh VP. Use of hyperbaric oxygen in traumatic brain injury: retrospective analysis of data of 20 patients treated at a tertiary care centre. Br J Neurosurg 2012;26:202–207. 22085249 10.3109/02688697.2011.626879

[16] Barret KF, Masel B, Patterson J, Scheibel RS, Corson KP, Mader JT. Regional CBF in chronic stable TBI treated with hyperbaric oxygen. Undersea Hyperb Med 2004;31:395–406. 15686271

[17] Rosenfeld JV, Maas AL, Bragge P, Morgantikossmann, Manley GT, Gruen RL. Early management of severe traumatic brain injury. Lancet 2012;380:1088–1098. 22998718 10.1016/S0140-6736(12)60864-2

[18] Godman CA, Chheda KP, Hightower LE, Perdrizet G, Shin DG, Giardina C. Hyperbaric oxygen induces a cytoprotective and angiogenic response in human microvascular endothelial cells. Cell Stress Chaperones 2010;15:431–432. 19949909 10.1007/s12192-009-0159-0 PMC3082642

[19] Efrati S, Fishlev G, Bechor Y, et al. Hyperbaric oxygen induces late neuroplasticity in post stroke patients-randomized, prospective trial. PLoS One 2013;8:e53716. 23335971 10.1371/journal.pone.0053716 PMC3546039

[20] Rahav BG, Haim G, Gregori F, et al. Hyperbaric oxygen therapy can lmprove post concussion syndrome years after mild traumatic brain injury-randomized prospective trial. PLoS One 2013;8:e79995. 24260334 10.1371/journal.pone.0079995 PMC3829860

[21] Wang S, Cheng H, Dai G, et al. Umbilical cord mesenchymal stem cell transplantation significantly improves neurological function in patients with sequelae of traumatic brain injury. Brain Res 2013;1532:76–78. 23942181 10.1016/j.brainres.2013.08.001

[22] Andelic N, Bautz-Holter E, Ronning PA, et al. Does an early onset and continuous chain of rehabilitation improve the long-term functional outcome of patients with severe traumatic brain injury? J Neurotrauma 2012;29:66–74. 21864138 10.1089/neu.2011.1811

[23] Walker WC, Franke LM, Cifu DX, Hart BB. Randomized, sham-controlled, feasibility trial of hyperbaric oxygen for service members with postconcussion syndrome: cognitive and psychomotor outcomes 1 week postintervention. Neurorehabil Neural Repair 2013;28:420–432. 24370568 10.1177/1545968313516869

[24] Cifu DX, Walker WC, West SL, et al. Hyperbaric oxygen for blast-related postconcussion syndrome: three-month outcomes. Ann Neurol 2014;75:277–286. 24255008 10.1002/ana.24067

[25] Wolf EG, Prye J, Michaelson R, Brower G, Profenna L, Boneta O. Hyperbaric side effects in a traumatic brain injury randomized clinical trial. Undersea Hyperb Med 2012;39:1075–1082. 23342764

[26] Harch PG, Neubauer RA, Hyperbaric oxygen therapy in global cerebral ischemia/anoxia and coma. In: Jain KK, ed. Chapter 19, in Textbook of Hyperbaric Medicine, 5th revised ed. Seattle: Hogrefe and Huber Publishers, 2009:235–274.

[27] Lin JW, Tsai JT, Lee LM, et al. Effect of hyperbaric oxygen on patients with traumatic brain injury. Acta Neurochir 2008;Suppl. 101:145–149. 10.1007/978-3-211-78205-7_25 18642650

[28] Huang L, Obenaus A. Hyperbaric oxygen therapy for traumatic brain injury. Med Gas Res 2011;1:21–27. 22146562 10.1186/2045-9912-1-21 PMC3231802

[29] Rockswold SB, Rockswold GL, Zaun DA, Liu J. A prospective, randomized Phase II clinical trial to evaluate the effect of combined hyperbaric and normobaric hyperoxia on cerebral metabolism, intracranial pressure, oxygen toxicity, and clinical outcome in severe traumatic brain injury. J Neurosurg 2013;118:1317–1328. 23510092 10.3171/2013.2.JNS121468 PMC12928550

[30] Lund VE, Kentala E, Scheinin H, et al. Effect of age and repeated hyperbaric oxygen treatments on vagal tone. Undersea Hyperb Med 2005;32:111–119. 15926303

[31] Camporesi EM. Side effects of hyperbaric oxygen therapy. Undersea Hyperb Med 2014;41:253–257. 24984321

[32] Katz D, Polyak M, Coughlan D, Meline N, Roche A. Natural history of recovery from brain injury after prolonged disorders of consciousness: outcome of patients admitted to inpatient rehabilitation with 1 to 4 year follow-up. Arch Phys Med Rehabil 2009;177:73–88. 10.1016/S0079-6123(09)17707-5 19818896

[33] Innes KE, Selfe TK, Alexander GK, Taylor AG. A new educational film control for use in studies of active mind-body therapies: acceptability and feasibility. J Altern Complement Med 2011;17:453–458. 21554109 10.1089/acm.2010.0401 PMC4209488

[34] Chin LM, Chan L, Woolstenhulme JG, Christensen EJ, Shenouda CN, Keyser RE. Improved cardiorespiratory fitness with aerobic exercise training in individuals with traumatic brain injury. J Head Trauma Rehabil 2014;30:382–390. 10.1097/HTR.0000000000000062 PMC4685937 24901330

[35] Schwandt M, Harris JE, Thomas S, Keightley M, Snaiderman A, Colantonio A. Feasibility and effect of aerobic exercise for lowering depressive symptoms among individuals with traumatic brain injury: a pilot study. J Head Trauma Rehabil 2012;27:99–103. 21386712 10.1097/HTR.0b013e31820e6858

[36] Swain RA, Berggren KL, Kerr AL, Patel A, Peplinski C, Sikorski AM. On aerobic exercise and behavioral and neural plasticity. Brain Sci 2012;2:709–744. 24961267 10.3390/brainsci2040709 PMC4061809

